# Ohio USA stoneflies (Insecta, Plecoptera): species richness estimation, distribution of functional niche traits, drainage affiliations, and relationships to other states

**DOI:** 10.3897/zookeys.178.2616

**Published:** 2012-03-29

**Authors:** R. Edward DeWalt, Yong Cao, Tari Tweddale, Scott A. Grubbs, Leon Hinz, Massimo Pessino, Jason L. Robinson

**Affiliations:** 1 University of Illinois, Prairie Research Institute, Illinois Natural History Survey, 1816 S Oak St., Champaign, IL 61820; 2 Western Kentucky University, Department of Biology and Center for Biodiversity Studies, Thompson Complex North Wing 107, Bowling Green, KY 42101

**Keywords:** Plecoptera, Stonefly, Biodiversity, Checklist, Ohio, North America

## Abstract

Ohio is an eastern USA state that historically was >70% covered in upland and mixed coniferous forest; about 60% of it glaciated by the Wisconsinan glacial episode. Its stonefly fauna has been studied in piecemeal fashion until now. The assemblage of Ohio stoneflies was assessed from over 4,000 records accumulated from 18 institutions, new collections, and trusted literature sources. Species richness totaled 102 with estimators Chao2 and ICE Mean predicting 105.6 and 106.4, respectively. Singletons and doubletons totaled 18 species. All North American families were represented with Perlidae accounted for the highest number of species at 34. The family Peltoperlidae contributed a single species. Most species had univoltine–fast life cycles with the vast majority emerging in summer, although there was a significant component of winter stoneflies. Nine United States Geological Survey hierarchical drainage units level 6 (HUC6) were used to stratify specimen data. Species richness was significantly related to the number of unique HUC6 locations, but there was no relationship with HUC6 drainage area. A nonparametric multidimensional scaling analysis found that larger HUC6s in the western part of the state had similar assemblages with lower species richness that were found to align with more savanna and wetland habitat. Other drainages having richer assemblages were aligned with upland deciduous and mixed coniferous forests of the east and south where slopes were higher. The Ohio assemblage was most similar to the well–studied fauna of Indiana (88 spp.) and Kentucky (108 spp.), two neighboring states. Many rare species and several high quality stream reaches should be considered for greater protection.

## Introduction

Regional biodiversity studies are of great importance for setting conservation priorities, in determining conservation status of species, and in examining factors that govern diversity (de Silva and Medellín 2001). The resulting species lists help other professionals to know what species live in the region. This is especially important for ecologists who use species and assemblage characteristics as water quality indicators. Conservation agencies can use these checklists and ecological relationships to help prioritize conservation initiatives, including the rehabilitation of habitat, purchase of land, planning for reintroductions, and establishment of imperilment risk for various taxa.

**Figure 1.**
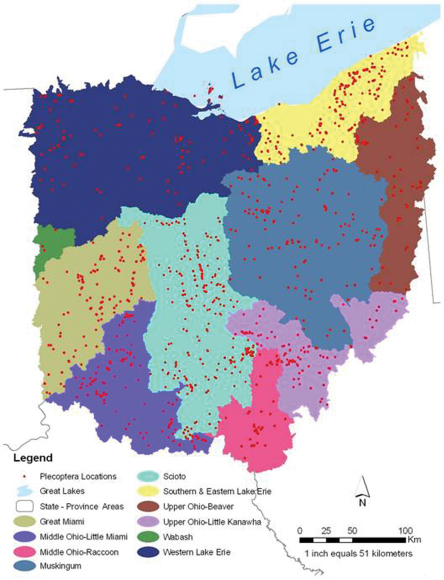
HUC6 drainages and point locations for Ohio Plecoptera collections.

**Figure 2.**
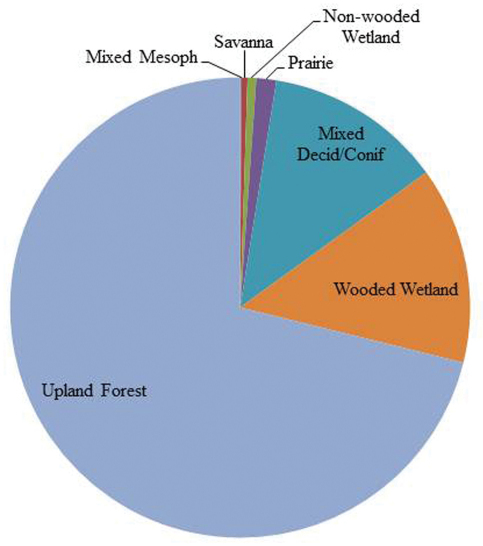
Pre-European settlement vegetation percentage cover for Ohio (from Ohio Department of Natural Resources 2003).

Ohio is an eastern state of the USA with a total area of 105,910 km^2^. It is bound on the south and east by the Ohio River and drained by 10 United States Geological Survey six digit scale Hydrologic Drainage Units (USDA 2009, HUC6s) (Fig. 1). The river and its tributaries have served as conduits for westward and northward migration from Kentucky, Pennsylvania, and West Virginia after the most recent glacial event cleared much of the fauna from the state. The Wisconsinan glaciation retreated 18,000 years bp, leaving a highly modified landscape. Areas in the northwest two–thirds of the state were heavily glaciated, leaving till (west central), lake (NW), and drift (NE) plain landscapes. Southeast of this line is found the unglaciated Allegheny plateau, an area of deeply incised hills and valleys filled with glacial outwash. Pre-European settlement land cover in Ohio was dominated by upland forest (71%), wooded wetland (13.8%), and mixed deciduous and coniferous forest (Fig. 2), the overall similarity to the Appalachian forests diminishing rapidly westward. Ohio’s human population in 2010 was 11,536,504 (USCB 2011).

Plecoptera (stoneflies) are known the world over as being environmentally sensitive (Fochetti and Tierno de Figueroa 2008). Because of this, they have been used extensively as indicators of water quality (Lenat 1993, Stewart and Stark 2002). Imperilment of significant numbers of species has been demonstrated in Europe (Zwick 1992), in the United States (Master et al. 2000), and for the USA state of Illinois (DeWalt et al. 2005).

Ohio’s stonefly fauna has been studied in a piecemeal fashion. Walker (1947) provided the first list of species, but covered only southeastern Ohio. Very few of his specimens survive today and most records were published only at the county level. Shortly thereafter, Gaufin (1956) updated the list from material collected from the southwestern quarter of the state. Many of his specimens were donated to the Monte L. Bean Museum at Brigham Young University. Several studies of a narrower scope were published for individual streams in northeastern Ohio in the 1970s and 1980s. Tkac and Foote (1978) published on the fauna of Stebbins Gulch in Geauga County, while Robertson (1979, 1984) published on Penitentiary Glen of Lake County. Fishbeck (1987) reported on stoneflies inhabiting Gray’s Run of Mahoning County and Beckett (1987) studied the nymphs of stoneflies inhabiting the polluted Ohio River at Cincinnati, Ohio. Additional records have appeared in several recent works including Stark (2004), Surdick (2004), Kondratieff (2004), and Kondratieff and Kirchner (2009). The last large undertaking was conducted by Tkac (1979) as part of his unpublished doctoral dissertation on the northeastern Ohio fauna. Relatively few Tkac specimens have been located; some may be in the United States National Museum.

The coauthors have embarked on a study of the stonefly fauna of the Midwest, including distribution modeling of up to 160 species known from Illinois, Indiana, Michigan, Ohio, Ontario, and Wisconsin in order to reconstruct pre-European settlement range. Given that there has been no comprehensive assessment of the stonefly assemblage in Ohio we have elected to prepare one. This analysis is based on the accumulated specimen records from our own efforts, the efforts of colleagues over several decades, the examination of nearly 30,000 specimens borrowed from regional museums, and reliable literature records. We ask several questions of these data:

• How many stonefly species inhabit Ohio?

• How completely has the fauna been sampled?

• How are functional niche traits distributed across the assemblage?

• Does drainage affiliation affect assemblage composition and species richness?

## Methods

### Specimen Records

Specimens are the only resource where identifications may be verified, so the study was based on an abundance of specimens examined from 18 regional museums (Table 1). Pinned specimens were relaxed in a humid chamber and the terminalia cleared in 10% KOH. Cleared terminalia were acidified in dilute acetic acid, rinsed in water, and stored in glycerin under the specimens. Eggs were removed before clearing and rehydrated to view ultrastructural characters. Eggs were also stored in glycerin below the specimen. Adults in alcohol were dealt with similarly, the eggs and abdomens stored in shell vials inside the larger vial. Each specimen or series in a vial was associated with a database record using a paper catalog number. Label data and carefully scrutinized literature records were added to a local database. The raw data set is available as “Appendix 1 Raw Specimen data” in Excel Spreadsheet format. A glossary to Appendix 1 has also been provided as supplementary data.

**Table 1.** Specimen origin, institutional coden, number of specimen records, and number of specimens examined.

Institution	Coden	#Records	Specimens
Brigham Young University	BYU	1167	18811
B. P. Stark Collection	BPSC	6	81
Canadian National Collection	CNC	46	252
Cincinnati Museum of Natural History	CMNH	2	2
Cleveland Museum Natural History	CLEV	66	171
Field Museum Natural History, Chicago	FMNH	13	40
Illinois Natural History Survey	INHS	639	2839
Michigan State University	MSUC	11	63
Ohio Biological Survey	OBS	573	2690
Ohio Environmental Protection Agency	OEPA	83	142
Ohio Historical Society	OHSC	17	17
Ohio State University	OSU	468	668
Purdue University	PURC	7	18
R. Fred Kirchner Collection	RFKC	164	857
Royal Ontario Museum	ROME	3	15
Southern Illinois University Carbondale	SIUC	1	5
University of Michigan	UMMZ	3	3
Western Kentucky University	WKU	170	873
Literature	Author Year	641	4940
Total		4,080	32,487

New specimens were collected using sweep nets, beating sheets, hand picking, and dipnetting throughout the state. Many nymphs were reared in Styrofoam cups or in a Frigid Units Living Stream at the University of Illinois. Illinois Natural History Survey (INHS) specimens collected after 2007 were preserved in 95% EtOH and stored in a –20C freezer for future molecular studies.

Locations for all specimens were georeferenced to the finest scale permitted by the label data. Coordinate precision for each record was marked as a radius about the location: from GPS = code 1(10 m); post–processed with small town or road crossing and stream name = code 2 (1,000 m); town name only or large town with stream name = code 3(10,000 m), county level record = code 4 (100,000 m); state level record = code 5 (1,000,000) m. Only records with codes 1–3 were used for species accumulation curves and nonparametric multidimensional scaling (NMDS) analyses.

### Data Analysis

**How many stonefly species inhabit Ohio and adequacy of sampling?**These are complex questions that we answered in two ways. First, a list of species was tallied from all specimen data. All data were used for this purpose. Second, records with precision code 1–3 were used to estimate species richness using the program EstimateS v8.2 (Colwell 2006). Museum data are biased due to the inability to quantify the sampling effort for every researcher. To reduce this bias, we elected to use unique locations as sample units, regardless of date of collection. In this way, the number of sites with multiple visits is maximized. This is important for stoneflies due to their proclivity for a succession of species throughout the year and because the nymphs for many species are unidentifiable (Stewart and Stark 2002). Unique locations are presented as “Appendix 2 OH Unique Locations”. Raw data were summarized to produce a species presence/absence by unique location data matrix, available as “Appendix 3 spp. Location Matrix”. The data matrix was analyzed using 50 randomizations, strong hash encryption, and randomization without replacement. Cumulative assemblage richness (Sobs–Mao Tau), the number of singletons and doubletons, and two estimators of species richness (Chao2 and ICE Mean) were plotted versus unique locations. Comparison of species richness and assemblage composition with Illinois, Indiana, Kentucky, Michigan, Ontario, Pennsylvania, Wisconsin, and West Virginia was conducted by compiling species lists from these states using only published records compiled in DeWalt et al. (2011) and Nelson (2008). A species by state/province matrix was constructed and a Sørensen Index of Similarity of each pairwise comparison calculated using EstimateS (Colwell 2006).

**How are species traits distributed across the assemblage?** Poff et al. (2006) provided a summary of functional niche traits for several life history, mobility, morphological, and ecological traits for aquatic insects. These were expressed at family and generic taxonomic levels and are useful in characterizing large scale ecological and evolutionary conditions under which a species or an assemblage lives. Stonefly functional niche traits are perhaps the best known of all aquatic insects in North America (see vast summaries of Stewart and Stark 2002 and references therein). We compiled a subset of Poff et al. (2006) functional niche traits and, to the best of our ability, recorded trait states for all species known to occur in Ohio (Table 2). These trait states were drawn from numerous literature sources (Harper 1973a, b, Harper and Hynes 1972, MacKay 1969, Stewart and Stark 2002) and from 30 years of professional experience working in the Midwest region. Of course, life histories and detailed feeding studies were not available for all species, so trait states for many species were surmised from closely related taxa. The number of species thought to have each trait state was tallied for the entire Ohio assemblage.

**Table 2.** Ohio stoneflies. Stream widths inhabiting and functional niche traits. Width 1=seep, 2=1–2 m, 3=3–10 m, 4=10–30 m, 5=30–60 m, 6=>60 m, 7=Lake Erie. Voltinism 1, 2 or 3 yr; development 1=fast, 2=slow seasonal. Diapause 1=present, 2=absent. Dispersal Season W=winter, Sp=spring, Su=summer. Feeding O=omnivore, P=predator, S=shredder. Female Mobility L=low, M=moderate, H=high. Nymphal Growth=months of growth, Respiration 1=no gills, 2=with gills. Size at maturity 1=<9 mm, 2=9–16 mm, 3=>16 mm. Emergence Synchrony 1=>1 mo., 2=<1 mo. Thermal preference 1=coldwater, 2=coolwater, 3=warmwater. Active hyperlinks are embedded LSIDs linking to species pages in the Plecoptera Species File website (DeWalt et al. 2012).

**Species**	**Species Niche Traits**											
	**Stream Width**	**Voltinism**	**Development**	**Diapause**	**Dispersal Season**	**Feeding**	**Fem. Mobility**	**Nymphal Growth**	**Respiration**	**Size at Maturity**	**Synchrony**	**Thermal Pref.**
**Capniidae**												
*Allocapnia forbesi* Frison	1–5	1	1	1	W	S	L	6	1	1	1	2
*Allocapnia frisoni* Ross & Ricker	2–3	1	1	1	W	S	L	6	1	1	1	2
*Allocapnia granulata* Claassen	3–6	1	1	1	W	S	L	6	1	1	1	3
*Allocapnia illinoensis* Frison	1–4	1	1	1	W	S	L	6	1	1	1	2
*Allocapnia indianae* Ricker	3–4	1	1	1	W	S	L	6	1	1	1	2
*Allocapnia mystica* Frison	2–4	1	1	1	W	S	L	6	1	1	1	2
*Allocapnia nivicola* (Fitch)	2–5	1	1	1	W	S	L	6	1	1	1	2
*Allocapnia ohioensis* Ross & Ricker	1–4	1	1	1	W	S	L	6	1	1	1	2
*Allocapnia pechumani* Ross & Ricker	3	1	1	1	W	S	L	6	1	1	1	2
*Allocapnia pygmaea*( Burmeister)	3	1	1	1	W	S	L	6	1	1	1	2
*Allocapnia recta* (Claassen)	2–5	1	1	1	W	S	L	6	1	1	1	2
*Allocapnia rickeri* Frison	2–5	1	1	1	W	S	L	6	1	1	1	2
*Allocapnia smithi* Ross & Ricker	2	1	1	1	W	S	L	6	1	1	1	2
*Allocapnia vivipara* (Claassen)	1–6	1	1	1	W	S	L	6	1	1	1	3
*Allocapnia zola* Ricker	3	1	1	1	W	S	L	6	1	1	1	2
*Paracapnia angulata* Hanson	1–4	1	2	2	Sp	S	M	11	1	1	2	1
**Leuctridae**												
*Leuctra alexanderi* Hanson	1–2	1	2	2	Su	S	M	11	1	1	1	2
*Leuctra duplicata* Claassen	3	1	2	2	Su	S	M	11	1	1	2	2
*Leuctra ferruginea* (Walker)	2–4	1	2	2	Su	S	M	11	1	1	2	2
*Leuctra rickeri* James	1–4	1	1	1	Sp	S	M	6	1	1	1	2
*Leuctra sibleyi* Claassen	2–4	1	1	1	Sp	S	M	6	1	1	1	2
*Leuctra tenella* Provancher	2	1	2	2	Su	S	M	11	1	1	2	2
*Leuctra tenuis* (Pictet)	2–4	1	1	2	Su	S	M	6	1	1	1	2
*Paraleuctra sara* (Claassen)	1–4	1	1	1	Sp	S	M	6	1	1	2	2
*Zealeuctra claasseni* (Frison)	2–3	1	1	1	W	S	M	6	1	1	1	2
*Zealeuctra fraxina* Ricker & Ross	2–3	1	1	1	W	S	M	6	1	1	1	2
**Nemouridae**												
*Amphinemura delosa* (Ricker)	1–5	1	1	1	Sp	S	M	6	2	1	1	2
*Amphinemura nigritta* (Provancher)	2–4	1	2	1	Su	S	M	9	2	1	1	1
*Amphinemura varshava* (Ricker)	1–6	1	1	1	Sp	S	M	6	2	1	1	2
*Nemoura trispinosa* Claassen	1–3	1	2	2	Su	S	M	11	1	1	1	1
*Ostrocerca albidipennis* (Walker)	2–3	1	1	1	Sp	S	M	6	1	1	1	1
*Ostrocerca truncata* (Claassen)	2–3	1	1	1	Sp	S	M	6	1	1	1	1
*Prostoia completa* (Walker)	2–3	1	1	1	Sp	S	M	6	1	1	2	2
*Prostoia similis* (Hagen)	2–3	1	1	1	Sp	S	M	6	1	1	2	2
*Soyedina vallicularia* (Wu)	1–3	1	1	1	W	S	M	11	1	1	1	1
**Taeniopterygidae**												
*Strophopteryx fasciata* (Burmeister)	3–6	1	1	1	W	S	M	6	1	2	1	2
*Taeniopteryx burksi* Ricker & Ross	2–6	1	1	1	W	S	M	6	2	2	1	3
*Taeniopteryx lita* Frison	6	1	1	1	W	S	M	6	2	2	1	3
*Taeniopteryx maura* (Pictet)	2–5	1	1	1	W	S	M	6	2	2	1	3
*Taeniopteryx metequi* Ricker & Ross	3–5	1	1	1	W	S	M	6	2	2	1	2
*Taeniopteryx nivalis* Fitch	3–5	1	1	1	W	S	M	6	2	2	1	2
*Taeniopteryx parvula* Banks	3–5	1	1	1	W	S	M	6	2	2	1	2
**Chloroperlidae**												
*Alloperla caudata* Frison	2–4	1	1	1	Sp	P	M	6	1	1	2	2
*Alloperla chloris* Frison	2–4	1	2	2	Sp	P	M	11	1	1	2	1
*Alloperla idei* Ricker	3	1	2	2	Sp	P	M	11	1	1	2	1
*Alloperla imbecilla* (Say)	2–3	1	2	2	Sp	P	M	11	1	1	2	1
*Alloperla neglecta* Frison	3	1	2	2	Sp	P	M	11	1	1	2	1
*Alloperla petasata* Surdick	2–3	1	1	2	Sp	P	M	6	1	1	2	2
*Alloperla usa* Ricker	2–4	1	2	2	Sp	P	M	11	1	1	2	1
*Haploperla brevis* (Banks)	1–3	1	1	1	Sp	P	M	6	1	1	2	2
*Sweltsa hoffmani* Kondratieff & Kirchner	1–5	1	2	2	Sp	P	M	11	1	1	2	1
*Sweltsa lateralis* (Banks)	3	1	2	2	Sp	P	M	11	1	1	2	1
**Perlidae**												
*Acroneuria abnormis* (Newman)	3–6	2	2	2	Su	P	H	23	2	3	2	2
*Acroneuria carolinensis* (Banks)	2–5	2	2	2	Su	P	H	23	2	3	2	2
*Acroneuria covelli* Grubbs & Stark	5	2	2	2	Su	P	H	23	2	3	2	2
*Acroneuria evoluta* Klapálek	4–6	2	2	2	Su	P	H	23	2	3	2	3
*Acroneuria filicis* Frison	2–6	2	2	2	Su	P	H	23	2	3	2	2
*Acroneuria frisoni* Stark & Brown	2–7	1	2	2	Su	P	H	11	2	3	2	2
*Acroneuria internata* (Walker)	4–6	2	2	2	Su	P	H	23	2	3	2	2
*Acroneuria kosztarabi* Kondratieff & Kirchner	3	2	2	2	Su	P	H	23	2	3	2	2
*Acroneuria lycorias* (Newman)	2	2	2	2	Su	P	H	23	2	3	2	2
*Acroneuria perplexa* Frison	3–6	2	2	2	Su	P	H	23	2	3	2	3
*Agnetina annulipes* (Hagen)	4	2	2	2	Su	P	H	23	2	3	2	2
*Agnetina capitata* (Pictet)	2–5	2	2	2	Su	P	H	23	2	3	2	2
*Agnetina flavescens* (Walsh)	2–6	2	2	2	Su	P	H	23	2	3	2	2
*Attaneuria ruralis* (Hagen)	4	2	2	2	Su	P	H	23	2	3	2	3
*Eccoptura xanthenes* (Newman)	2	2	2	2	Su	P	H	23	2	3	1	1
*Neoperla catharae* Stark & Baumann	3–5	1	2	2	Su	P	H	11	2	2	2	2
*Neoperla clymene* (Newman)	3–5	1	2	2	Su	P	H	11	2	2	2	3
*Neoperla coosa* Stark & Smith	3–5	1	2	2	Su	P	H	11	2	2	2	2
*Neoperla gaufini* Stark & Baumann	3–6	1	2	2	Su	P	H	11	2	2	2	2
*Neoperla mainensis* Banks	4–7	1	2	2	Su	P	H	11	2	2	2	3
*Neoperla occipitalis* (Pictet)	3–6	1	2	2	Su	P	H	11	2	2	2	2
*Neoperla robisoni* Poulton & Stewart	3–5	1	2	2	Su	P	H	11	2	2	2	2
*Neoperla stewarti* Stark & Baumann	2–6	1	2	2	Su	P	H	11	2	2	2	2
*Paragnetina media* (Walker)	3–5	2	2	2	Su	P	H	23	2	3	2	2
*Perlesta adena* Stark	2–6	1	1	1	Su	P	H	4	2	2	2	2
*Perlesta decipiens*(Walsh)	3–6	1	1	1	Su	P	H	4	2	2	2	3
*Perlesta golconda* DeWalt & Stark	3	1	1	1	Su	P	H	4	2	2	2	3
*Perlesta lagoi* Stark	3–6	1	1	1	Su	P	H	4	2	2	2	3
*Perlesta shubuta* Stark	3–6	1	1	1	Su	P	H	4	2	2	2	2
*Perlesta teaysia* Kondratieff & Kirchner	2–5	1	1	1	Su	P	H	4	2	2	2	2
*Perlesta xube* Stark & Rhodes	4	1	1	1	Su	P	H	4	2	2	2	2
*Perlesta* sp. I–4	3-6	1	1	1	Su	P	H	4	2	2	2	2
*Perlinella drymo* (Newman)	3–5	1	1	1	Su	P	H	9	2	3	2	2
*Perlinella ephyre* (Newman)	3–6	1	1	1	Su	P	H	9	2	2	2	3
**Perlodidae**												
*Clioperla clio* (Newman)	1–5	1	1	1	Sp	P	H	9	1	2	2	2
*Cultus decisus decisus* (Walker)	2–3	1	2	2	Sp	P	H	11	1	2	2	1
*Diploperla robusta* Stark & Gaufin	1–5	1	1	1	Sp	P	M	6	1	2	2	2
*Isoperla bilineata* (Say)	3–6	1	1	1	Su	P	H	6	1	2	2	3
*Isoperla burksi* Frison	2	1	1	1	Sp	P	M	6	1	2	2	2
*Isoperla decepta* Frison	2–4	1	1	1	Sp	O	M	6	1	2	2	2
*Isoperla dicala* Frison	2	1	2	2	Su	P	H	11	1	2	2	2
*Isoperla holochlora* (Klapálek)	3	1	2	2	Su	P	H	11	1	2	2	1
*Isoperla montana* (Banks)	2–4	1	2	2	Sp	P	M	11	1	2	2	2
*Isoperla nana* (Walsh)	2–5	1	1	1	Sp	P	M	6	1	1	2	3
*Isoperla signata* (Banks)	2–4	1	2	2	Su	P	H	11	1	2	2	2
*Isoperla transmarina* (Newman)	2–4	1	2	2	Sp	P	H	11	1	2	2	2
*Malirekus* cf. *iroquois* Stark & Szczytko	3	1	2	2	Su	P	H	11	1	3	2	1
**Peltoperlidae**												
*Peltoperla arcuata* Needham	2–3	1	2	2	Su	S	H	11	2	2	2	1
**Pteronarcyidae**												
*Pteronarcys* cf. *biloba* Newman	3	3	1	1	Su	S	H	35	2	3	2	1
*Pteronarcys dorsata* (Say)	5–6	3	1	1	Su	S	H	35	2	3	2	2

**Does drainage affiliation affect assemblage composition and species richness?** HUC6 units were used to stratify the records (U.S. Department of Agriculture 2009) (Fig. 1). These hydrologic units drain areas of markedly different topography and glacial history and were the smallest hierarchical drainage unit for which the data would support subdivision. The smallest subunit, the Wabash, was dropped from the analysis because it had only three records and a single species represented. Two indices of sample intensity for each HUC6 drainage were calculated by dividing the number of records and unique locations by the drainage area in km^2^. Relationships between species richness, the number of unique locations, and drainage area and the SQRT of area were investigated using regression analysis as provided within the statistical program R version 2.14.0.

The NMDS analysis was conducted using PC–ORD Ver. 5 (PC–ORD 2011) to determine if there were relationships between HUC6 assemblages and a suite of environmental variables. A species presence/absence data matrix was constructed by HUC6 for the nine drainages. The matrix is available as “Appendix 4 spp. vs HUC6s”. A second data set of 16 environmental variables was constructed that consisted of percentage pre-European settlement vegetation data (Ohio Department of Natural Resources 2003) and elevation, relief ratio, and slope variables (U.S. Geological Survey 2008). These data are included in Table 3.

**Table 3.** Nine Ohio HUC6 drainages, number of unique locations, species richness and 16 environmental variables used in NMDS analysis. Pre-European settlement vegetation is percentage cover. PEMM = Portage Escarpment Mesophytic Forest, Forest_UL =Upland forest, Forest_MX = Mixed deciduous/coniferous forest, WL_NW = nonwooded wetland, WL_W = wooded wetland, RR_Mean = Relief Ratio mean.

				% Pre-European Settlement Vegetation							Elevation (m)					Relief Ratio		
USGS<br/> HUC6s	Sites	Species Richness	DRAIN_km2X1000	PEMM	PRAIRIE	FOREST_UL	FOREST_MX	SAVANNA	WL_NW	WL_W	ELEV_MEAN	ELEV_STD	ELEV_MAX	ELEV_MIN	ELEV_MEDIAN	RR_MEAN	RR_MEDIAN	SLOPE %
WL_Erie	95	25	23.31	0.0	2.3	59.3	0.7	2.0	1.6	34.1	239	35.8	411	147	233	0.35	0.33	0.8
SE_LErie	133	65	8.25	1.2	0.1	68.8	24.0	0.0	1.0	4.9	285	53.9	420	173	285	0.45	0.45	2.2
UOH_Bvr	50	44	8.62	0.0	0.0	80.2	17.2	0.0	0.2	2.4	330	38.6	436	182	336	0.58	0.61	6.2
UOH_LKan	80	48	7.93	0.0	0.2	80.7	15.9	0.0	0.3	2.9	263	45.8	433	151	261	0.40	0.39	10.3
Musk	116	56	20.85	0.0	0.0	76.2	20.6	0.0	0.4	2.8	316	43.0	460	174	317	0.50	0.50	6.8
Scioto	201	71	16.87	0.0	3.9	69.9	12.1	0.0	0.3	13.7	283	43.2	454	142	289	0.45	0.47	3.9
GrMiami	109	34	10.70	0.0	1.3	79.4	5.1	0.0	0.4	13.8	299	44.7	469	138	306	0.49	0.51	1.9
MOH_Rac	39	35	5.06	0.0	0.0	84.9	10.0	0.0	0.0	5.1	233	34.1	363	136	234	0.43	0.43	9.8
MOH_LMia	119	57	9.37	0.0	1.8	60.8	16.4	0.0	0.0	21.0	269	45.5	404	131	275	0.50	0.53	4.6

## Results

### Species Richness and Community Composition

A total of 4,051 database records accounting for 32,487 specimens were accumulated for this project (Table 1). The museum that contributed the most records was the Bean Museum at Brigham Young University, followed by the INHS Insect Collection. Cumulatively, these records produced an Ohio stonefly assemblage of at least 102 species (Table 2) from 942 unique locations (Fig. 1, Appendix 2).

**Figure 3.**
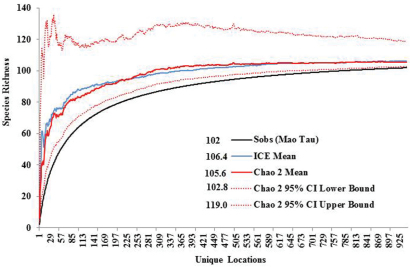
Ohio Plecoptera species richness, actual vs. predicted.

**Figure 4.**
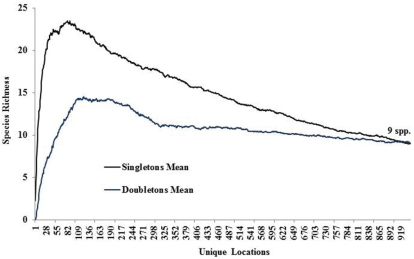
Singleton and doubleton species richness.

**Figure 5.**
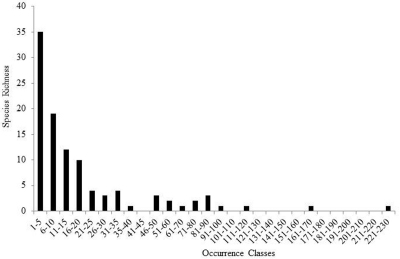
Species richness of Ohio Plecoptera in 5 increment occurrence classes.

Species richness estimators predicted slightly higher values (Fig. 3). The Chao2 estimator predicted 105.6 species with 95% confidence intervals (CI) ranging from 102.8 to 119.0 species. Another estimator, ICE Mean, produced a mean value of 106.4 (EstimateS does not provide CIs for this estimator). Many rare species were found. Singletons and doubletons accounted for 17.6% of all species (Fig. 4). Approximately 75% of all species were collected at fewer than 20 locations, while only three species were collected from over 100 locations: *Allocapnia vivipara* (Claassen) (223 sites), *Perlesta* cf. *lagoi* Stark (161 sites), and *Acroneuria frisoni* Stark & Brown (115 sites) (Fig. 5).

### Assemblage Composition

**Figure 6.**
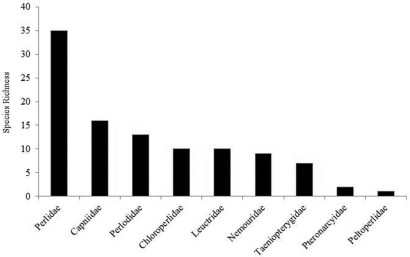
Species richness of Ohio Plecoptera families.

The stonefly fauna of Ohio was represented by all nine families known to inhabit the North American continent (Fig. 6). Perlidae contributed the greatest number species (32.4%). Capniidae, one of the winter stonefly families, contributed 15.7% of all species found. Four other families contributed between 8.8% and 12.7% of the fauna. Roach stoneflies, Peltoperlidae, contributed a single species, *Peltoperla arcuata* Needham.

Stream size is often an important determinant of stonefly communities. This dataset demonstrated that most species inhabited a range of stream sizes (Table 2). There were several species relegated to small streams. Five species occurred only in streams that were 1 to 2 m across: *Allocapnia smithi* Ross & Ricker, *Leuctra tenella* Provancher, *Eccoptura xanthenes* (Newman), and *Isoperla burksi* Frison. Several others inhabited streams 3 to 10 m across: *Allocapnia pechumani* Ross & Ricker, *Allocapnia zola* Ricker, *Leuctra duplicata* Claassen, *Acroneuria kosztarabi* Kondratieff & Kirchner, *Peltoperla arcuata* Needham, and *Malirekus* cf. i*roquois* Stark & Szczytko. While some species were most frequently collected from large rivers, they could be taken in somewhat smaller streams as well. Only two species were confirmed to inhabit Lake Erie, from several locations around the Bass Islands: both perlids, *Acroneuria frisoni* and *Neoperla mainensis* Banks. Both species have been extirpated from Lake Erie, but *Acroneuria frisoni* is frequently collected throughout all but the northwestern corner of the state.

### Distribution of Species Traits

The vast majority of stonefly species inhabiting Ohio have single year life cycles; only 17 (16.7%) had multiyear life cycles (Table 4). Species with fast–seasonal cycles, those with nymphal growth periods that lasted 4-9 months, are slightly more frequently collected than slow seasonal (growth 11, 23, 35 mo. = direct development) species. Species with egg or nymphal diapause were also more frequently collected in Ohio than were non–diapausing species. Most species dispersed in the summer, a trait state that was not surprising given the number of perlid species found. Ohio did have quite a large proportion of so called “winter stoneflies”, species that emerge in winter that belong to the families Capniidae and Taeniopterygidae.

The number of months of nymphal growth had the largest number of trait states of all species trait categories. There were 9 species with an exceedingly short growth period of four months. These included all *Perlesta* species, a genus where females lay eggs in June and July and the eggs diapause until March. The most frequent growth period was 6 months, accounting for 40.8% of all species. Only a few species exhibited a nine month growing period, *Perlinella*, *Clioperla* clio (Newman), and *Amphinemura nigritta* (Provancher), while a total of 31 exhibited 11 month growth periods. Growth periods lasting 23 (*Acroneuria*, *Agnetina*, *Attaneuria*, *Paragnetina*) and 35 (*Pteronarcys*) mos. were also present.

**Table 4.** Species traits distributions for the Ohio stonefly assemblage. Traits from Table 2.

Volt.		Devel.		Diap.		Dispers.		Feed.		Mobil.		Grow.		Respir.		Size (mm)		Synch. (mo.)		Therm.	
1	85	1	57	1	56	W	25	O	1	L	15	4	8	1	56	<9	46	>1	36	1	19
2	15	2	45	2	46	Sp	28	P	56	M	42	6	42	2	46	9–16	35	<1	66	2	67
3	2					Su	49	S	45	H	45	9	4			>16	21			3	16
												11	31								
												23	15								
												35	2								

More research on the feeding of Plecoptera nymphs and adults is necessary to effectively use functional feeding group designations for ecological research. The study of gut enzymatic activity (Tierno de Figueroa et al. 2011) and use of stable isotopes to determine nutrient sources (Miyasaka and Genkai-Kato 2009, Reynolds et al. 1997) has demonstrated how little we understand about the feeding of stoneflies. The functional feeding group designations presented herein must be considered tentative, but should be useful to characterize the distribution of feeding groups within Ohio. Predators were the most frequent functional feeding group found among Ohio species, a function of the dominance of perlids once again. The next largest feeding group was the shredders of tree leaves. One species has been listed as an omnivore, *Isoperla decepta* Frison (Perlodidae), since its lacinia have apical paired, chisel-like teeth that may indicate that scraping of periphyton is an option at least during part of its nymphal life. That it is a perlodid suggests that it might also eat animal matter. It is probable that when detailed enzymatic or stable isotope studies are conducted on stoneflies, many species will be viewed as omnivorous, at least during part of their nymphal growth.

Female mobility is an important trait that confers ability to colonize and recolonize after local extinction. The vast majority of species exhibited medium to high female mobility. Low female dispersal ability was exhibited by 14.6% of Ohio species. Low mobility is a complex character state that is exhibited mostly by winter emerging species that emigrate by crawling, skating (wings held up to breeze and skating on tarsi), or floating on logs or ice floes.

The presence or absence of gills is often thought of as indicative of a species’ ability to tolerate warmer waters and lower oxygen concentrations. No formal analysis has been conducted of the association of gilled stoneflies with water temperature preference, but informal studies in Europe indicate that many gilled species inhabit mountainous areas with cold and cool water temperatures (M. Tierno de Figueroa pers. comm.). In eastern North America there are over 50 species of perlids (*Attaneuria*, *Perlesta*, *Perlinella*, *Neoperla*, *Acroneuria*, *Agnetina*, and *Paragnetina*) that tolerate warmer streams and often dominate the stonefly assemblage at low elevation.

The majority of Ohio species (54.9%) did not have gills (some Perlodidae with submental gills were counted as gill-less). Most of these species lived in cool and coldwater streams or lived in streams as nymphs only during the colder times of the year (e.g., they have diapause that restricts nymphs to winter or spring season). A total of 45.1% of species used gills. The vast majority of these were species in the family Perlidae.

Size at maturity is a trait that has direct bearing on risk to survival. Larger species are usually longer lived and exposed to risks for a longer period of time and may make more attractive prey items than some smaller species. Small species (<9 mm total length) were much more frequent in the list than large species (>16 mm). Smaller species are more likely to have short growth periods and diapause. They are also more likely to have fast cycles and disperse in the winter and spring than larger species.

Species with synchronous (<1 month) emergence periods were more frequent in Ohio than asynchronous (>1 month) species. Winter emerging species tend to have less synchronous emergence periods due to fluctuating winter temperatures from freezing and thawing. Those that emerge in spring and summer have sharper seasonal cues that lead to nymphal development being more synchronous, leading to emergence of adults over a shorter period of time. Spring and summer emerging species were three times more frequent in Ohio than winter-emerging ones; hence, the great disparity in synchronous over asynchronous emergence is easily understood.

Thermal requirements are not well understood in stoneflies or other aquatic insects and the terms coldwater, coolwater, and warmwater are relative when it comes to defining a temperature requirement. Trait state assignment here is based more on professional experience than for any other set of traits. Coldwater species contribute only 18.6% to the total number of species found in OH. Most of Ohio is or was heavily wooded, a feature usually related to coolwater conditions. This is by far the most frequent thermal tolerance state for stoneflies in Ohio. Another 15.7% of species can truly be categorized as warmwater species. This would include several perlid species.

**Figure 7.**
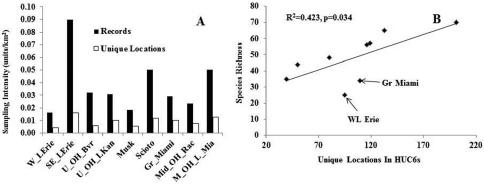
**A–B** Sampling intensity, drainage area, unique locations, and species richness relationships for HUC6 drainages **A** Sampling intensity for HUC6 drainages **B** Species richness vs. number of unique locations in HUC6 drainage areas.

**Does drainage affiliation affect assemblage composition and species richness?** Two measures of sample intensity across the nine HUC6 drainages demonstrated that three HUC6s were most heavily sampled: Southern and Eastern Lake Erie, Scioto, and Middle Ohio and Little Miami drainages (Fig. 7A). The two largest drainages, Western Lake Erie and Muskingum appeared to be somewhat under-sampled compared to other drainages. Across all drainages, species richness was significantly related to the number of unique locations sampled (R^2^=0.42, p=0.03, n=9, Fig. 7B). Western Lake Erie and Great Miami drainages had lower than predicted species richness. These are the western-most drainages in the state with a landscape composed of flatter till and lake plains, possibly accounting for lower species richness. There was no significant relationship between species richness and drainage area (R^2^=0.0, p=0.87, n=9) or the square root of drainage area (R^2^=0.0, p=0.97, n=9).

**Figure 8.**
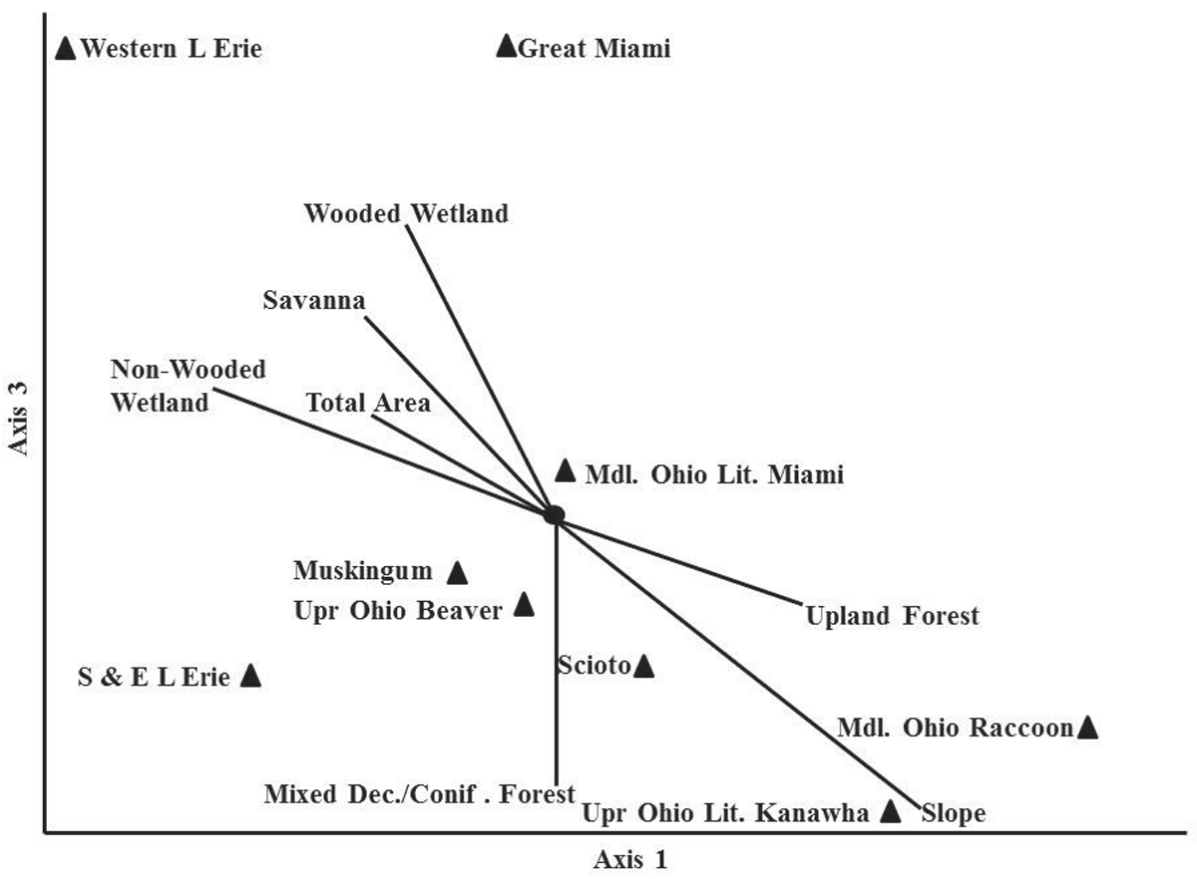
Non–parametric Multi–Dimensional Scaling of Ohio Plecoptera assemblages associated with HUC6 drainages. Axis 1 vs Axis 3.

Randomization tests in the NMDS analysis recommended a three dimensional solution. An overall stress value for the three dimensional analysis was low at 1.53. A plot of Axis 1 vs Axis 3 separated the communities of Ohio stoneflies best (Fig. 8). The western–most HUC6 drainages (Western Lake Erie and Great Miami) were separated from all other assemblages by being strongly associated with wetland and savanna pre-European settlement land covers. There was a lesser association with large total area as well. These two drainages supported the lowest species richness. Centrally located in the plot were five HUC6 communities that associated with low percentage wetland and savanna coverage and with smaller drainage sizes. These drainages had much richer stonefly communities and an association with upland hardwoods. Two other HUC6 assemblages (Upper Ohio Little Kanawha and Middle Ohio Raccoon) were separated from the others by being associated with a high percentage of mixed deciduous/coniferous forest and high percentage slope.

## Discussion

### Comparison to assemblages found in nearby states/provinces

**Figure 9.**
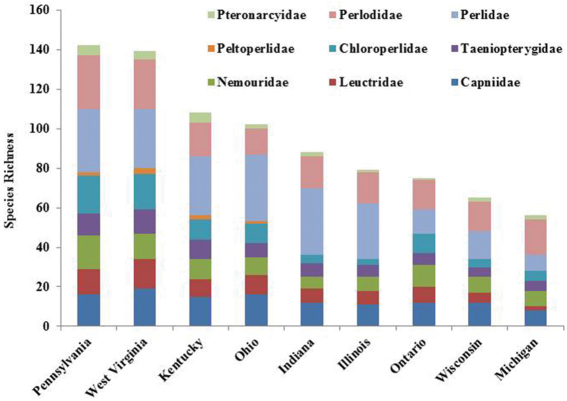
Comparison of Ohio Plecoptera assemblage with Midwest states/provinces.

Ohio is a state that has its eastern flank in the Allegheny Plateau, an extension of the Appalachian Mountains. Ohio’s western flank is mostly till plain resulting from the Wisconsinan glaciation. The stonefly fauna found in the state is a mixture of species requiring cooler waters and deep forest and those that have evolved with warmwater streams and even intermittency of flow. The number of species occurring in Ohio is indicative of being between these two extremes. Ohio supports at least 102 species, maybe as high as 119 (e.g., Chao 2 upper 95 percentile) (Fig. 4). Pennsylvania to the east supports 39.2% more species than Ohio, with West Virginia having 36.3% more species. It appears that a continuous drop of species occurs westward and northward from Ohio (Fig. 9). The northward decline of species occurs as species usually inhabiting unglaciated terrain drop out. Sørensen’s Index of Similarity suggested that Kentucky and Indiana assemblages have the greatest similarity with the Ohio assemblage, rather than the more mountainous states of Pennsylvania and West Virginia (Table 5). The lowest similarities were observed with more recently glaciated Michigan, Ontario, and Wisconsin.

**Table 5.** Sørensen Index of Similarity between Plecoptera assemblages for nearby states/provinces in relation to Ohio. Codes are IL=Illinois, IN=Indiana, MI=Michigan, OH=Ohio, ON=Ontario, WI=Wisconsin, PA=Pennsylsvania, WV=West Virginia, and KY=Kentucky.

	IL	IN	MI	OH	ON	WI	PA	WV	KY
IL	0								
IN	0.814	0							
MI	0.444	0.417	0						
OH	0.659	0.743	0.415	0					
ON	0.416	0.429	0.656	0.551	0				
WI	0.569	0.523	0.744	0.548	0.686	0			
PA	0.425	0.470	0.394	0.629	0.535	0.512	0		
WV	0.440	0.485	0.349	0.628	0.467	0.461	0.826	0	
KY	0.588	0.684	0.354	0.749	0.437	0.474	0.648	0.721	0

### Rare species

Ohio had 18 species that were collected from just one or two locations, 17.6% of all species found. A discussion for a limited number of these species is presented below to identify for state, federal, and non–profit conservation organizations that these species, and the streams in which they reside, should be considered for protected status. Some streams are already in the public trust.

*Allocapnia indianae* Ricker. A large series was collected by W. E. Ricker (1952) on 19 March 1950 from three locations in Scioto Co.: W Portsmouth, Turkey Creek, 38.69690, -83.10076; W. Portsmouth, road 25, Odell Creek, 38.70286, -83.11518; Rd. 125, 9 mi. E Blue Creek, Turkey Creek, 38.727194, -83.17265. The species in known from scattered locations in unglaciated areas of Indiana, Kentucky, and Ohio, but has also been collected in the Finger Lakes region of New York (Ross and Ricker 1971).

*Allocapnia smithi* Ross & Ricker. This species was taken from a single location in Warren Co., 10 km ESE Lebanon, Randall Run, Fort Ancient State Memorial, 39.40951, -84.09039, 12 February 1966, H. B. Cunningham. This species is known from scattered locations in unglaciated sections of Alabama, Illinois, Indiana, Kentucky, and Ohio (DeWalt and Grubbs 2011, Ross and Ricker 1971).

*Leuctra duplicata* Claassen. This species was taken from two small streams adjacent to each other in Ashtabula Co.: 4.5 km NNW Hartsgrove at Callahan Rd., Crooked Creek, 41.64210, -80.97250, 3 June 1997, R. W. Baumann & B. C. Kondratieff, 7 males, 9 females; same location, 2 June 1989, R. W. Baumann & R. F. Kirchner, 2 M; Callahan Road, spring fed tributary Crooked Creek, 41.64245, -80.97374, 2 June 1989, R. W. Baumann & R. F. Kirchner, 42 males, 28 females. Crooked Creek is a darkly stained, 3–m wide stream that holds other coolwater species such as *Acroneuria carolinensis* (Banks), *Isoperla* cf. *montana* (Banks), *Soyedina vallicularia* (Wu), *Paracapnia angulata* Hanson, *Allocapnia rickeri* Frisonр and *Taeniopteryx metequi* Ricker & Ross. This species is known from much of northeastern North America, but Ohio is the furthest west it has been collected (DeWalt et al. 2011).

*Alloperla idei* Ricker. This species is represented by two records in the Allegheny Plateau region of SE OH. Lawrence Co., 17 km SSE Oak Hill, Buffalo Creek, Wayne National Forest, 38.74598, -82.54445, 27 May 2010, S. A. Grubbs, 3 males, 9 Females; Pickaway Co. Laurelville, Tributary Laurel Run, 39.47390, -82.74263. 23 May 1953, [A. R. Gaufin], 2 males. This species occurs throughout much of eastern Canada and the eastern USA (Baumann and Kondratieff 2009, DeWalt et al. 2011).

*Alloperla neglecta* Frison. This species was collected as a single adult male from Lake Co., at Paine Road, Leroy Township, Paine Creek, Paine Falls Metropolitan Park, 41.71669, -81.14356, 31 May 1975, M. K. Tkac. Tkac (1979) considered this a relict population in a pristine stream. The nearest known populations of this species are in North Carolina, Tennessee, and Virginia (DeWalt et al. 2011).

*Sweltsa lateralis* (Banks). Several adults were collected from Mahoning Co., Lowellville, Grays Run, 41.04353, -80.53957, May 1985, D. W. Fishbeck. This Gray’s Run was recorded as supporting several other species of Appalachian Mountains or coldwater habitat.

*Peltoperla arcuata* Needham. This species was once thought to be exceedingly rare in Ohio, but it has been located in four counties now in 1 to 2–m wide coldwater, ravine streams, usually associated with mixed deciduous and coniferous (hemlock and white pine) forest. This is the only representative of the family Peltoperlidae to inhabit Ohio. Loss of this species would remove an entire family of stoneflies from the state. Locations include: Ashland Co., Tributary to Hog Hollow Creek, Big Lyon Falls Creek, Little Lyon Falls Creek, Tributary Clear Fork Mohican River (Hemlock Grove CG), all in Mohican State Park; Knox Co., Tributary Mohican River at Greer; Mahoning Co., Gray’s Run at Lowellville; Muskingum Co., Seep Tributary Wills Creek. The species is distributed over several eastern states and the Canadian province of Quebec (DeWalt et al. 2011).

*Isoperla burksi* Frison. Three nymphs were collected from Scioto Co., Mackletree Run, 12 km SSW West Portsmouth, Shawnee State Forest. 38.7236, -83.1821, 14 April 2006, R. E. DeWalt, S. K. Ferguson, R. F. Kirchner. This species has been collected from the Ozark Mountains to New Jersey, mostly in unglaciated habitats. Mackletree Run is a stream that typifies semi–permanent streams in unglaciated southern Ohio.

*Malirekus* cf. *iroquois* Stark & Szczytko. We have seen two nymphs from a single location in Monroe Co., Tributary Stillhouse Run, 39.78146, -80.85288. M. Leuhrs. Fishbeck (1987) reported *Malirekus hastatus* (Banks) from Gray’s Run. However, Stark and Szczytko (1988) described *Malirekus iroquois* from nearby states after Fishbeck’s work, so the identification of these specimens is in doubt. Nonetheless, this is a second location for *Malirekus* in Ohio and an additional Appalachian species in Grays Run.

*Pteronarcys* cf. *biloba* Newman. Nymphs with lateral extensions of the abdominal terga have been known from Ohio since Tkac’s (1979) dissertation, but no adults have been collected and the record was not published. Bolton (2010) published the first Ohio record for this Appalachian species, but from a second location. Ashtabula Co., Indian Creek, Montgomery Road, RM 1.3, 41.5644, -80.9328, 11 September 2007, M. J. Bolton, nymph; Lake Co., Kirtland Hills at Sperry Road, Pierson’s Creek, 41.62818, -81.31494, 5/11/1978, M. K. Tkac, nymph; same location, 5/20/1978, M. K. Tkac, nymph. These Ohio records are the furthest west for the species; it is found throughout northeastern North America as far south as northern Georgia (Nelson 1971).

### Streams with diverse assemblages

Several streams across the state are exceedingly rich in species and have been well sampled. A tributary of the East Branch of the Chagrin River in Stebbins Gulch of Holden Arboretum (Geauga Co.) produced 28 species including several coldwater species. A tributary of the East Fork Queer Creek at Ash Cave (Hocking Co.) has produced 23 species. The Olentangy River near Columbus (Franklin Co.) has produced 17 species historically. Upstream of the city near Highbank Metropark a diverse assemblage still persists, although it may not hold the full complement of species once found in the river. The Clear Fork of the Mohican River within the ravine area of Mohican State Park (Ashland Co.) has produced 14 species and probably still supports most of them. Big Lyons Falls Creek, also in Mohican State Park; a tributary of the North Fork Little Beaver River, 5 km S Negley, in Columbiana Co.; and Mill Creek at Doty Road (Lake Co.) all produced 13 species. Gray’s Run at Lowellville (Mahoning Co.) produced a total of 12 species, many of which were Appalachian coldwater species. Most of these locations are protected by public or private means. These, and several others too numerous to list here, are important for protecting the lotic diversity of aquatic organisms in the Ohio.

### HUC6 Drainages

HUC6 drainages explain some of the variation in stonefly communities across Ohio (Fig. 8). However, the stress value was relatively low for this analysis, suggesting that there may be better stratification systems and variables to explain Ohio species richness patterns. Additional classification systems that could be tested in future analyses include U. S. Environmental Protection Agency level III ecoregions and the Nature Conservancy’s Ecological Drainage Units.

The data suggested that smaller drainages of the eastern and southern part of the state followed a pattern of increasing richness with drainage area, but that the largest drainage did not (Fig. 7B). Western Lake Erie was under-sampled in relation to its area (Fig. 7A, B). However, this is not to say that the species richness in the drainage was suspiciously low. The Western Lake Erie assemblage was defined by wetland categories and large drainage area (Fig. 8). This basin has low topographic relief and fine substrates left behind by large glacial lakes. The low species richness there was most likely a function of the flatter landscape, low current velocity, and finer substrates. The Great Miami drainage just to the south of Western Lake Erie also had relatively low species richness. It too was defined by similar environmental conditions as Western Lake Erie, however, its southern end contains one coldwater stream (Mad River) and fast flowing reaches, which enhanced its species richness over that of Western Lake Erie. Indeed, the two drainages had a 61% Sørensen quotient of similarity. The Great Miami basin supported such cool- and coldwater species as *Agnetina capitata* (Pictet), *Paragnetina media* (Walker), *Leuctra tenuis* (Pictet), *Nemoura trispinosa* Claassenр and *Soyedina vallicularia* (Wu). Several species that require faster flowing, wooded streams were also found in the Great Miami and not the Western Lake Erie drainage.

Some of the most species rich HUC6 assemblages were located in a band of upland forest and higher gradient streams that straddled drift plain and unglaciated terrain from Cincinnati to Ashtabula. These assemblages were dominated by widespread species that typically inhabit cool and warmwater streams. There was also a small component of coldwater Appalachian fauna including species of *Ostrocerca*, *Amphinemura nigritta*, *Cultus decisus decisus*, *Malirekus* cf. *iroquois*, *Pteronarcys* cf. *biloba*, *Amphinemura neglecta*, *Alloperla lateralis*, *Alloperla idei*, and *Allocapnia pechumani*, among others. These species have found coldwater habitat in ravine streams of Southern and Eastern Lake Erie, Upper Ohio and Beaver, Scioto, and Muskingum drainages.

Two other drainages, the Upper Ohio Little Kanawha and the Middle Ohio Raccoon, had assemblages that were defined by mixed deciduous and coniferous forests and higher slope values in southern, unglaciated Ohio. These are relatively small drainages with short streams, many of which become intermittent in the summer. Consequently, many species with egg or nymphal diapause are found here. This trend also occurs in southern Illinois and Indiana (DeWalt et al. 2005, DeWalt and Grubbs 2011, Webb 2002).

### Changes in the fauna

The Plecoptera assemblage presented herein is largely of pre-European settlement nature from records spanning the late 1880s to present. Some changes are likely to have occurred in Ohio, just as they did in IL (DeWalt et al. 2005). Additional targeted sampling would be required to assess changes in detail. However, some changes are readily apparent, mostly the loss of the large perlid species that lived in larger rivers and had 11 or 23 month nymphal growth.

*Acroneuria abnormis* (Newman). Once found in moderately large to large rivers throughout the state, it is now only known from recent records in the Grand River (Lake Co.), Clear Fork Mohican River (Ashland Co.), and Ohio River (Clermont Co.).

*Acroneuria frisoni* Stark & Baumann. This species was found at 115 unique locations across the state, with many being from recent years. Many historical records of adults and nymphs were found for the Bass Islands in the Western Basin of Lake Erie. Unfortunately, of the 94 records from the islands, the most recent was for 1961. It has been extirpated from Lake Erie.

*Acroneuria filicis* Frison. It once inhabited several moderately sized drainages in the eastern half of the state. The only recent records are from the Grand River (Lake Co.), West Fork River (Brown Co.), and Ohio Brush Creek (Scioto Co.).

*Acroneuria perplexa* Frison. This species has been lost from moderately large and large rivers in the eastern half of Ohio. No records are available for over 50 years. It is probably extirpated from Ohio.

*Acroneuria evoluta* Klapálek: There is a single historical record from 1936 from Black Lick Creek near Columbus (Franklin Co.). It is probably extirpated from Ohio.

*Attaneuria ruralis* (Hagen). A male and female of this large species were collected from Columbus (Franklin Co.), either from the Olentangy or Scioto rivers, in 1925. It is probably extirpated from Ohio.

*Neoperla mainensis* Banks. This species was historically collected from Columbus (Franklin Co.), either from the Scioto or Olentangy rivers; the Clear Fork of the Mohican (Ashland Co.), and South Bass Island (Ottawa Co.) area of Lake Erie. It is probably extirpated from the state with none of the 38 records being more recent than 1922.

## Conclusions

A large dataset from 18 regional museums, new collecting, and trusted literature sources demonstrated that the Plecoptera assemblage of Ohio is rich with at least 102 species. All nine North American families were represented with Perlidae contributing 33% of all species (Fig. 6).

Ohio species are mostly univoltine-fast with egg or nymphal diapause and the largest proportion of them are summer emerging (Table 4). Nymphs are predominantly predatory, but a large number of species that shred conditioned leaves and wood were also present. The majority of species inhabited cool, forest-covered streams. The state has significant Appalachian elements, mostly within the ravine streams of central and eastern drainages associated with upland forests and mixed deciduous and coniferous forest habitats (Fig. 8). Despite this, the fauna of Ohio is most closely aligned with Indiana and Kentucky assemblages (Table 5).

It appears that Ohio has been well sampled; species estimators (Chao2 and ICE Mean) suggested that 3 or 4 more species could be found. Given that neighboring Pennsylvania and West Virginia have 142 and 139 species, respectively, it is likely that species shared with them will eventually be found in Ohio (Fig. 9). It is highly probably that these additional species will be located in ravine streams in eastern and southern Ohio.

A great number of species in Ohio were rare, being known from only 1–5 locations (Figs. 4 and 5). Many of these are candidates for protection, as are several stream reaches that support high numbers of species or assemblages that are rare. In addition, it appears that several species have experienced local or statewide extirpations. These have mostly been of univoltine and semivoltine species whose eggs hatch directly. This is consistent with losses reported for IL (DeWalt et al. 2005). Conservation organizations working in Ohio may download these data directly supplemental files.

This paper lays a foundation for planned future work, including natural range modeling of species within the larger framework of the Midwest USA and Canada. This will allow our research team to reconstruct pre-European settlement ranges for most species in the region. We are also focusing considerable effort to use these baseline distributions against which to measure climate related changes in distribution by modifying climate variables in light of predicted CO_2_ emissions scenarios.

## Appendices

Raw data and data matrices. (doi: 10.3897/zookeys.178.2616.app1) File format: MS Excel spreadsheet (xls).

**Explanation note:** Appendices include an Excel spreadsheet with all data used in analyses for this article: "Appendix1 Raw Specimen Data", "Glossary Appendix 1", "Appendix 2 OH Unique Locations", "Appendix 3 spp. Location Matrix", and "Appendix 4 spp. vs HUC6s".
